# Altered striatal endocannabinoid signaling in a transgenic mouse model of spinocerebellar ataxia type-3

**DOI:** 10.1371/journal.pone.0176521

**Published:** 2017-04-27

**Authors:** Carmen Rodríguez-Cueto, Mariluz Hernández-Gálvez, Cecilia J. Hillard, Patricia Maciel, Sara Valdeolivas, José A. Ramos, María Gómez-Ruiz, Javier Fernández-Ruiz

**Affiliations:** 1 Instituto Universitario de Investigación en Neuroquímica, Departamento de Bioquímica y Biología Molecular, Facultad de Medicina, Universidad Complutense, Madrid, Spain; 2 Centro de Investigación Biomédica en Red de Enfermedades Neurodegenerativas, Madrid, Spain; 3 Instituto Ramón y Cajal de Investigación Sanitaria, Madrid, Spain; 4 Departamento de Psicobiología, Facultad de Psicología, Universidad Complutense, Madrid, Spain; 5 Department of Pharmacology and Toxicology, Medical College of Wisconsin, Milwaukee, Wisconsin, United States of America; 6 Life and Health Sciences Research Institute (ICVS), School of Medicine, University of Minho, Braga, Portugal; 7 ICVS/3B’s-PT Government Associate Laboratory, Braga/Guimaraes, Portugal; University of Florida, UNITED STATES

## Abstract

Spinocerebellar ataxia type-3 (SCA-3) is the most prevalent autosomal dominant inherited ataxia. We recently found that the endocannabinoid system is altered in the *post-mortem* cerebellum of SCA-3 patients, and similar results were also found in the cerebellar and brainstem nuclei of a SCA-3 transgenic mouse model. Given that the neuropathology of SCA-3 is not restricted to these two brain regions but rather, it is also evident in other structures (e.g., the basal ganglia), we studied the possible changes to endocannabinoid signaling in the striatum of these transgenic mice. SCA-3 mutant mice suffer defects in motor coordination, balance and they have an abnormal gait, reflecting a cerebellar/brainstem neuropathology. However, they also show dystonia-like behavior (limb clasping) that may be related to the malfunction/deterioration of specific neurons in the striatum. Indeed, we found a loss of striatal projecting neurons in SCA-3 mutant mice, accompanied by a reduction in glial glutamate transporters that could potentially aggravate excitotoxic damage. In terms of endocannabinoid signaling, no changes in CB_2_ receptors were evident, yet an important reduction in CB_1_ receptors was detected by qPCR and immunostaining. The reduction in CB_1_ receptors was presumed to occur in striatal afferent and efferent neurons, also potentially aggravating excitotoxicity. We also measured the endocannabinoid lipids in the striatum and despite a marked increase in the FAAH enzyme in this area, no overall changes in these lipids were found. Collectively, these studies confirm that the striatal endocannabinoid system is altered in SCA-3 mutant mice, adding to the equivalent changes found in other strongly affected CNS structures in this type of ataxia (i.e.: the cerebellum and brainstem). These data open the way to search for drugs that might correct these changes.

## Introduction

Important changes in the endocannabinoid signaling system have been found in the CNS structures affected in most acute and chronic neurodegenerative disorders ([1,2] for review). Such changes have been interpreted in two ways. On the one hand, dysregulation of cannabinoid receptor type-1 (CB_1_) signaling has been associated with maladaptive responses that eventually contribute to disease symptoms (e.g., bradykinesia, ataxia, choreic movements) and/or to enhanced neuronal deterioration (e.g., heightened glutamate toxicity: [1,2]). Indeed, the elevated activity of endocannabinoid hydrolyzing enzymes like fatty acid amide hydrolase (FAAH) and monoacylglycerol lipase (MAGL) has been associated with such maladaptive responses, provoking excessive degradation of endocannabinoids that dampens their prosurvival properties ([2,3] for review). On the other hand, the up-regulation of cannabinoid receptor type-2 (CB_2_), particularly in activated glial elements, produces an endogenous protective response that helps limit the consequences of neurotoxic insults, mainly inflammatory events but also oxidative stress and excitotoxicity, all of which operate in most neurodegenerative disorders ([2,4,5] for review). A similar interpretation has been employed when considering the massive generation of endocannabinoids under neurotoxic conditions *in vitro* and *in vivo* ([2] for review).

In general, both dysregulation and endogenous protection are active in numerous neurodegenerative pathologies, both acute (e.g., stroke [6], brain trauma [7], spinal injury [8]) and chronic progressive disorders (e.g., Alzheimer’s disease [9], Parkinson’s disease [10], Huntington’s chorea [11,12], amyotrophic lateral sclerosis [13,14] and others [2]). Such responses have also been seen in autosomal-dominant spinocerebellar ataxias (SCAs; [15–17]), a group of inherited neurodegenerative disorders that mainly affect the cerebellum and its afferent/efferent structures, in which motor discoordination (“ataxia”) is the key symptom [18–21]. However, in most SCAs, and in particular SCA-3 or Machado-Joseph disease, the most prevalent of these rare disorders ([22] for review), neuronal malfunction/degeneration also occurs in extracerebellar structures like the brainstem, optic nerve, cortical areas and the basal ganglia ([23–25] for review). This explains why patients develop a broad-spectrum of neurological abnormalities, including primary cerebellar symptoms (e.g., progressive loss of motor coordination and abnormal gait: [24,26] for review) and non-cerebellar symptoms (e.g., muscle atrophy, oculomotor impairment, spasticity, and extrapyramidal signs such as rigidity and dystonia: [21,24,27] for review).

We recently found that endocannabinoid signaling appears to be dysregulated in the cerebellum SCA patients (including SCA-3), with important alterations to CB_1_ and CB_2_ receptors [15], and to FAAH and MAGL enzymes in *post-mortem* tissue [16]. Such alterations were also found recently in a transgenic mouse model of SCA-3 [17], which reproduces many of the neurological and neuropathological signs of the disease [28]. This earlier study [17] concentrated on two key CNS structures (cerebellum and brainstem) closely related to the classic symptoms (motor incoordination, imbalance, gait anomalies) and neuropathological lesions (the loss of specific neuronal subpopulations in the dentate nucleus, Purkinje layer and pontine nuclei) observed in SCA-3 [25,26,29,30]. Hence, the pharmacological correction of such changes in endocannabinoid signaling may represent a promising therapeutic option for this disease. However, the neuropathology of SCA-3 is not restricted to these two CNS areas and it is also affects other important structures like the basal ganglia, which explains the occurrence of extrapyramidal defects in SCA-3 patients [31] and in our SCA-3 transgenic mouse model (e.g., dystonia, rigidity: [17]). Therefore, the goal of this follow-up study was to investigate the changes to the endocannabinoid signaling system in the striatum of these transgenic mice using different biochemical and histological approaches.

## Materials and methods

### Animals and sampling

All experiments were carried out on pCMVMJD135 transgenic mice and their wild-type littermates [28]. Offspring were genotyped to detect those that carried the transgene containing the ataxin-3 mutation (around 135Q), as described previously [28]. All animals were housed under a controlled photoperiod (08:00–20:00 lights on) and temperature (22 ± 1°C), with free access to standard food and water. All the experiments were conducted in accordance with local and European legislation (directive 2010/63/EU), and following the principles of the ARRIVE guidelines. The protocols were also approved by the animal experimentation committee at our university (ref. CEA-UCM 56/2012).

We used non-transgenic and pCMVMJD135Q transgenic male mice for longitudinal studies to initially characterize the appearance of striatal malfunctioning/degeneration at the biochemical and histological levels. Subsequently possible alterations in the endocannabinoid signaling were evaluated in this structure. These animals were characterized 7–55 weeks after birth [17], detecting dystonic-like behavior (limb clasping) and establishing three different disease states: (i) an early symptomatic stage, up to 16 weeks after birth, characterized by a loss of muscle strength in the hanging wire test [28]; (ii) a stable symptomatic stage, up to 32 weeks after birth, involving impaired performance on the balance beam and rotarod indicative of impaired balance and motor coordination [28]; and (iii) an advanced stage, up to 56 weeks after birth, in which marked deficiencies in gait and dystonia were reflected by the animal performance in footprinting tests and through their clasping behavior, respectively [17].

Groups of SCA-3 transgenic and wild-type mice of these three ages were perfused transcardially with saline followed by fresh 4% paraformaldehyde in 0.1M phosphate buffered saline (PBS, pH 7.4), and their brain was collected and post-fixed for one day at 4°C. The tissue was then cryoprotected by immersion in a 30% sucrose solution for a further day, and finally stored at -80°C for Nissl staining and immunohistochemical analysis. The striatum was dissected out of brain of other mice and rapidly frozen on dry ice for subsequent biochemical analysis (qRT-PCR and western blotting), as well as to analyze the endocannabinoid concentrations. In all of the experiments, 6–8 male mice were used *per* experimental group.

### Biochemical analyses

#### Real time qRT-PCR analysis

Total RNA was extracted from the striatum using the SurePrep^™^ RNA/Protein Purification kit (Fisher BioReagents, Fair Lawn, NJ, USA) and it was quantified by spectrometry at 260 nm. The RNA purity was evaluated through the absorbance ratio at 260 and 280 nm, whereas its integrity was confirmed in agarose gels. Genomic DNA was removed enzymatically and single-stranded complementary DNA was synthesized from 1 μg of total RNA using a commercial kit (Rneasy Mini Quantitect Reverse Transcription, Qiagen, Izasa, Madrid, Spain). The cDNAs were amplified by quantitative real-time PCR using TaqMan Gene Expression Assays (Applied Biosystems, Foster City, CA, USA: see Table 1), using GADPH as an endogenous control gene for normalization. The PCR assays were performed using the 7300 Fast Real-Time PCR System (Applied Biosystems, Foster City, CA, USA) and the threshold cycle (Ct) was calculated using the instrument’s software (7300 Fast System, Applied Biosystems, Foster City, CA, USA).

**Table 1:** TaqMan probes used.

Gene Name	TaqMan Ref.
CB_1_ receptor	Mm00432621_s1
CB_2_ receptor	Mm00438286_m1
FAAH	Mm00515684_m1
MAGL	Mm00449274_m1
DAGL	Mm00813830_m1
NAPE-PLD	Mm00724596_m1
calbindin	Mm00486647_m1
NSE	Mm00469062_m1
BDNF	Mm01334042_m1
DARPP32	Mm00454892_m1
IL-1β	Mm00434228_m1
TNF-α	Mm99999068_m1
GLAST	Mm00600697_m1
GLT-1	Mm00441457_m1
GAPDH	Mm99999915_g1

#### Western blotting

Purified protein fractions were isolated using the SurePrep^™^ RNA/Protein Purification kit (Fisher BioReagents, Fair Lawn, NJ, USA) and aliquots of protein (20 μg) were boiled for 5 min in Laemmli SDS loading buffer (10% glycerol, 5% SDS, 5% β-mercaptoethanol, 0.01% bromophenol blue and 125 mM TRIS-HCl [pH 6.8]) prior to loading onto a 12% acrylamide gel (Bio-Rad Laboratories, Hercules, CA, USA). The proteins were transferred to a PVDF membrane (Immobilon-P, Millipore, Bedford, MA, USA) using a mini Trans-Blot Electrophoretic transfer cell (Bio-Rad Laboratories, Hercules, CA, USA), which were then blocked with 5% non-fat milk. The membranes were probed overnight at 4°C with the anti-FAAH antibody (see Table 2) and then for 2 hours at room temperature with an ECL^™^ Horseradish Peroxidase-linked whole secondary antibody (GE Healthcare UK Limited, Buckinghamshire, UK) diluted 1:5000. Reactive bands were detected by chemiluminescence with the Amersham^™^ ECL^™^ Prime Western Blotting Detection Reagent (GE Healthcare UK Limited, Buckinghamshire, UK) and the membranes were analyzed on a ChemiDoc station with Quantity one software (Bio-Rad Laboratories, Madrid, Spain). The optical density of the specific protein bands were assessed relative to the housekeeping protein GAPDH, and they were normalized to the controls.

**Table 2:** List of antibodies used in western blotting or immunohistochemical analyses (WB: Western blotting; IHC: Immunohistochemistry).

ANTIBODY	DILUTION (IHC)	DILUTION (WB)	CLASS	MANUFACTURER
anti-CB_1_: Rb-Af380	1:500		Polyclonal	Frontier Institute, Hokkaido, Japan
anti-FAAH:sc-26427	1:200	1:200	Polyclonal	Santa Cruz Biotechnology, CA, USA
anti-GAPDH:G8795		1:5000	Monoclonal	Sigma Chem., Madrid, Spain
anti-GFAP:Z0334	1:500		Polyclonal	DakoCytomation, Glostrup, Denmark
anti-Iba1:019–19741	1:300		Polyclonal	Wako, Osaka, Japan

#### LC/MS analysis for endocannabinoid levels

The amounts of endocannabinoids, and their related *N*-acylethanolamines and 2-acylglycerols, were determined by liquid chromatography-atmospheric pressure chemical ionization-mass spectrometry (LC/MS; 1100 LC-MSD, SL model; Agilent Technologies Inc., Wilmington, DE, USA), incorporating slight modifications to a previously described protocol [32]. Tissue samples were weighed and placed into borosilicate glass tubes containing 2 ml of acetonitrile with 84 pmol of [^2^H_8_]AEA and 186 pmol of [^2^H_8_]2-AG (Cayman Chemical, Ann Arbor, MI, USA). The tissues were homogenized with a glass rod and sonicated for 60 min. The samples were incubated overnight at -20°C to precipitate the proteins, they were centrifuged at 1,500g for 3 min, and the supernatant was removed to a new glass tube and evaporated to dryness under nitrogen. The samples were then resuspended in methanol (300 μl) to recapture any lipids adhering to the glass tube and dried again. Finally, the lipid extracts were suspended in 30 μl of methanol, 5 μl of which was used for the LC/MS analysis. Each sample was separated on a reverse-phase C18 column (Kromasil, 250 × 2 mm, 5-μm diameter) using mobile phase A (deionized water with 1 mM ammonium acetate and 0.005% acetic acid) and mobile phase B (methanol with 1 mM ammonium acetate and 0.005% acetic acid). The samples were eluted at a flow rate of 300 μl/min under a linear gradient, with the percentage of solvent B increasing linearly from 85% to 100% over 25 min and maintaining 100% solvent B for 10 min. Over the next 10 min, solvent B decreased linearly from 100 to 85% and it was held at 85% for an additional 10 min.

MS detection was carried out in the selected ion monitoring mode using m/z values to detect [^2^H_8_]AEA (m/z 356), AEA (m/z 348), [^2^H_8_]2-AG and 1(3)-AG (m/z 387), 2-AG and 1(3)-AG (m/z 379), PEA (m/z 299), OEA (m/z 326) and 2-OG and 1(3)-OG (m/z 357). As 2-AG and 2-OG are usually observed as doublets due to isomerization to the 1,3 form during extraction, the area of both peaks was combined to yield the total 2-AG or 2-OG. To calibrate the system, standard curves for the AEA, PEA, OEA, 2-AG and 2-OG (Cayman Chemical, Ann Arbor, MI, USA) were used. Each endocannabinoid concentration in the different samples was calculated by comparison with the corresponding deuterated internal standard: [^2^H_8_]AEA to calculate the AEA, PEA and OEA levels; and [^2^H_8_]2-AG in the case of 2-AG and 2-OG. The amounts of endocannabinoids and related *N*-acylethanolamines and 2-acylglycerols were normalized to the wet tissue weight and expressed as a percentages of the controls.

### Histological procedures

#### Tissue slices

Sagittal cryostat sections of fixed brains (30 μm thick) were collected on gelatin-coated slides, and either Nissl-stained or used for immunohistochemistry.

#### Nissl staining

Frozen sagittal brain sections were incubated with 0.1% methylene blue for 5 min and washed with distilled water. The sections were then dehydrated, sealed and coverslipped with non-aqueous mounting medium. A Leica DMRB microscope (Leica, Wetzlar, Germany) and a DFC300FX camera (Leica) were used to study and photograph the tissue, respectively. To count the number of Nissl-stained cells, high resolution photomicrographs were taken with a 20x objective under the same conditions of light, brightness and contrast. Four images from at least 3 sections per animal were analyzed to establish the mean of all the animals studied from each group. The data are expressed relative to the wild-type animals.

#### Immunohistochemistry

Sections were preincubated for 20 min in 0.1M PBS with 0.1% Triton X-100 [pH 7.4], and endogenous peroxidase activity was blocked by incubating them for 1 hour at room temperature in blocking solution (Dako Cytomation, Glostrup, Denmark). After incubation overnight at 4°C with the corresponding primary antibody (see Table 2) diluted in 0.1M PBS with 0.01% Triton X-100 [pH 7.4], the sections were washed in 0.1M PBS and incubated for 2 hours at room temperature with the appropriate biotin-conjugated anti-goat or anti-rabbit secondary antibodies (1:200; Vector Laboratories, Burlingame, CA, USA). An avidin-biotin complex (Vectastain^®^ Elite ABC kit; Vector Laboratories, Burlingame, CA, USA) and a DAB substrate–chromogen system (Dako Cytomation, Glostrup, Denmark) were used to obtain a visible reaction product. Negative control sections were obtained using the same protocol with the omission of the primary antibody. All sections for each immunohistochemical procedure were processed at the same time and under the same conditions. The sections were observed and photographed on a Leica DMRB microscope and with a Leica DFC300FX camera (Leica, Wetzlar, Germany). To quantify the immunoreactivity, the NIH Image Processing and Analysis software was used (ImageJ; NIH, Bethesda, MD, USA) on 4–5 randomly selected sections. The tissue was visualized with 20x objective by researchers blind to the experimental treatments and the data were expressed relative to the wild-type tissue.

### Statistics

The data were assessed using the GraphPad software (version 5.0), applying an unpaired Student’s t-test or two-way ANOVA for repeated measures followed by the Bonferroni test, as required.

## Results

### Characterization of the striatal damage in SCA-3 transgenic mice

**Fig 1:**
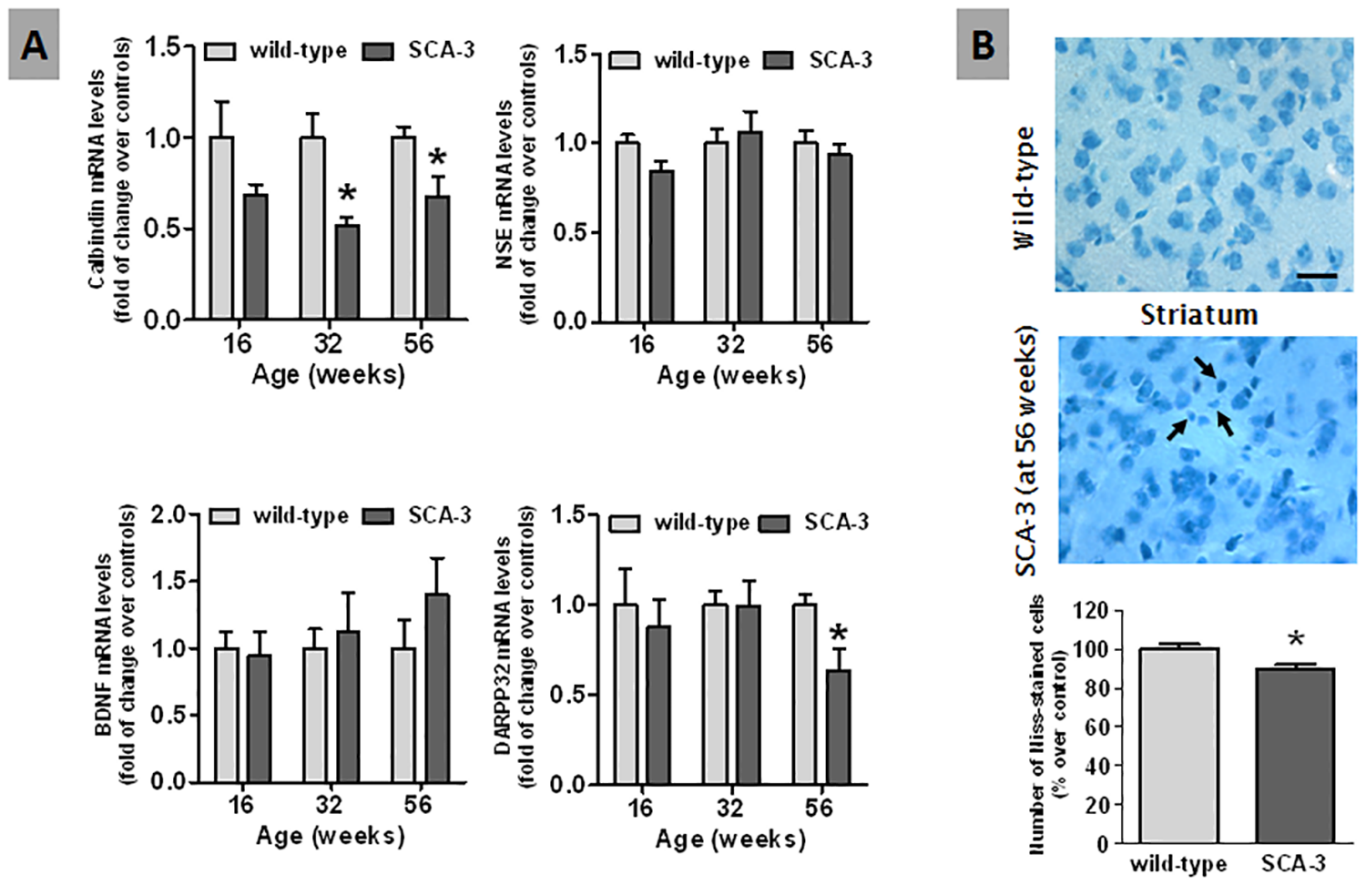
Histological and biochemical analysis of neuronal damage in the striatum of SCA-3 transgenic and wild-type mice. Panel A: A qPCR analysis of calbindin, neuron-specific enolase, BDNF and the striatal neuronal marker DARPP-32 expression in the striatum of SCA-3 transgenic and wild-type mice of different ages. Panel B: Nissl staining and neuron quantification in the striatum of SCA-3 transgenic and wild-type mice 56 weeks after birth (bar = 25 μm). Arrows indicate the presence of Nissl-stained cells with smaller cell bodies in the SCA-3 transgenic mice. In all cases, the values are expressed as the mean ± SEM of more than 6 animals *per* group, assessing the data using a Student’s t-test: *p<0.05 *versus* wild-type animals.

We recently described the progressive motor deterioration experienced by SCA-3 transgenic mice, as evident in the hanging wire, balance beam and rotarod tests [17,28]. In these studies, we also detected the presence of hindlimb clasping, a common marker of dystonia in mouse models of neurological disorders (including certain cerebellar ataxias [21]) and an extremely frequent response in SCA-3 mouse models [17,33]. Dystonia is an extrapyramidal sign often related to basal ganglia malfunction [34,35]. Thus, we have attempted to find a neuropathological basis for this response by analyzing the striatum of SCA-3 transgenic mice, a brain region significantly altered in SCA-3 patients [21]. We first assessed different biochemical markers that reflect either neuronal malfunction or neurodegeneration, combining qPCR with histological procedures to define neuronal loss or glial activation in this structure. Our qPCR studies revealed a marked reduction in the expression of the calbindin gene that appeared to commence at 16 weeks but that was significantly weaker at 32 and 56 weeks (Fig 1A). A decrease in DARPP32 expression was also seen at 56 weeks, a selective marker for striatal projection neurons, whereas other less-selective neuronal markers like NSE and BDNF did not appear to be affected (Fig 1A). The number of Nissl-stained cells in the striatal parenchyma of 56 week-old SCA-3 transgenic mice was also lower than in wild-type animals, and the surviving Nissl-stained cells in these mutant mice generally had a smaller cell body (see arrows in Fig 1B). Collectively, these data confirmed the deterioration of striatal neurons, in particular projection neurons, in SCA-3 transgenic mice, as also described in other SCA-3 models [33]. These data were consistent with the atrophy of the striatum and its output nuclei (globus pallidus, substantia nigra) described in SCA-3 patients [31].

**Fig 2:**
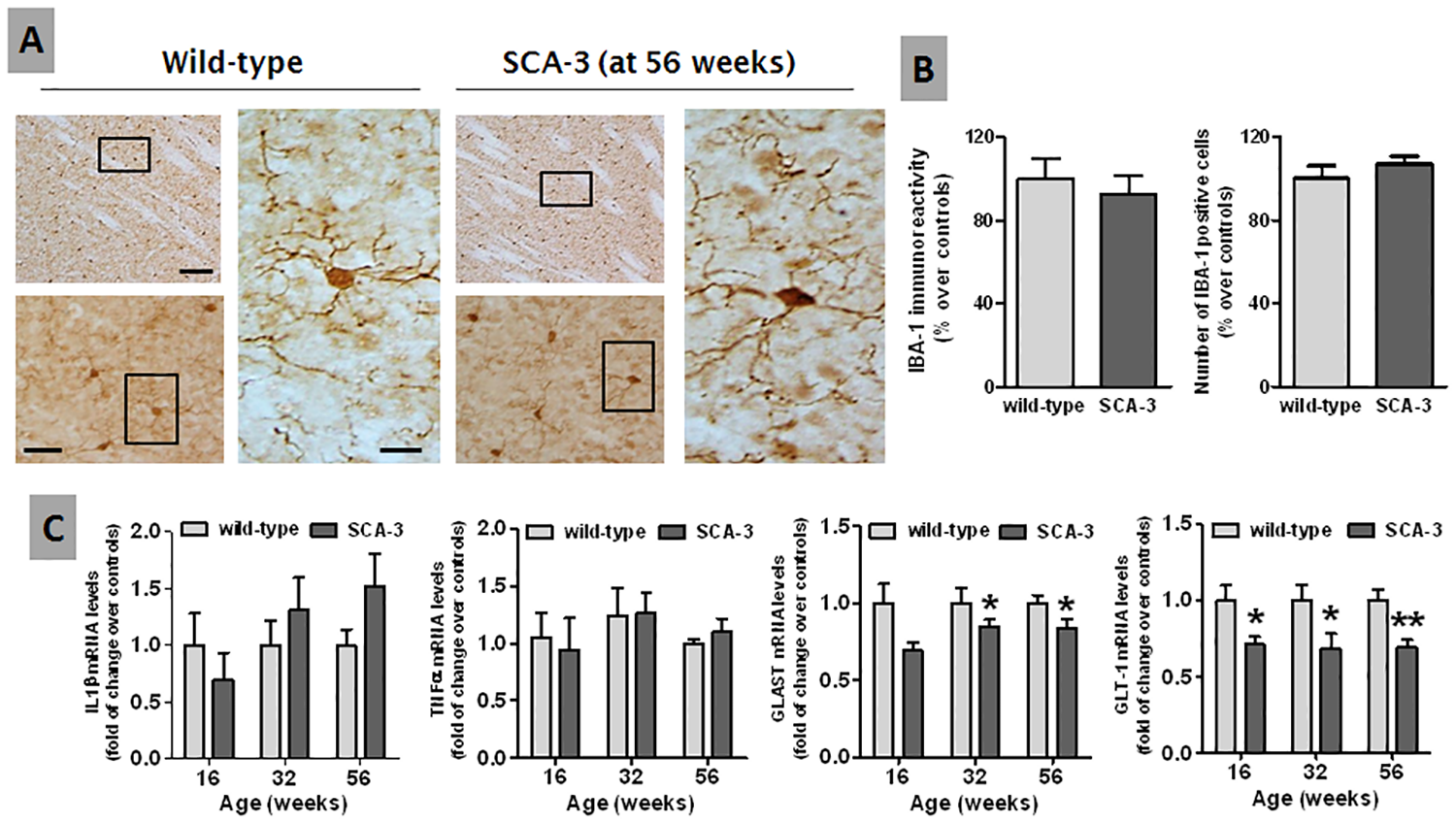
Histological and biochemical analysis of glial activation in the striatum of SCA-3 transgenic and wild-type mice. Panels A and B: Immunostaining (representative microphotographs and their quantification) for Iba-1 as a microglial marker in the striatum of 56 week-old SCA-3 transgenic and wild-type mice (bar = 200 μm, 50 μm and 20 μm, respectively). Panel C: qPCR analysis of the proinflammatory cytokines IL-1β and TNF-α, and of the glial glutamate transporters GLAST and GLT-1, in the striatum of SCA-3 transgenic and wild-type mice at different ages. In all cases, the values are expressed as the mean ± SEM of more than 6 animals *per* group, assessing the data using a Student’s t-test: *p<0.05, **p<0.01 *versus* wild-type animals.

We also investigated the possible glial activation in this structure using Iba-1 immunostaining to label microglial cells (Fig 2A). However, there were no significant differences in the number of Iba-1 positive cells or the staining intensity between 56 week-old (Fig 2B) or younger (data not shown) SCA-3 transgenic and wild-type mice. Similarly no such changes were evident in the expression of the proinflammatory cytokines IL-1β and TNF-α (Fig 2C). By contrast, the expression of glial glutamate transporters was significantly lower in the striatum of SCA-3 transgenic mice, both that of GLT-1 (16, 32 and 56 weeks) and GLAST (32 and 56 weeks: Fig 2C). However, the reduction in glutamate transporters was not associated with changes in GFAP immunostaining in the striatal parenchyma of SCA-3 transgenic mice relative to the wild-type animals (data not shown), a marker of astrocytes.

### Analysis of the endocannabinoid signaling system in the striatum of SCA-3 transgenic mice

**Fig 3:**
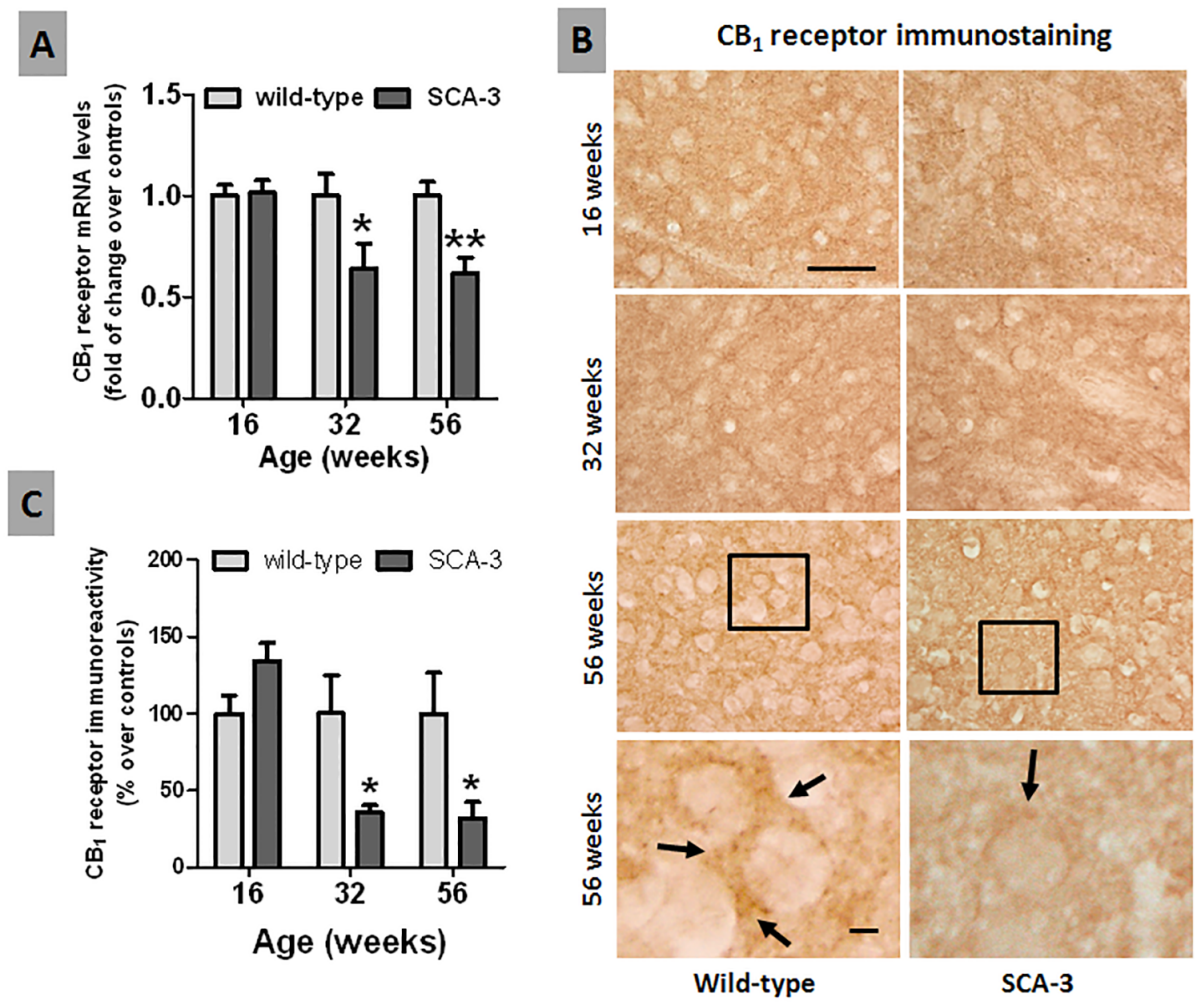
Histological and biochemical analysis of the CB_1_ receptor in the striatum of SCA-3 transgenic and wild-type mice. Panel A: qPCR analysis of the CB_1_ receptor in the striatum of SCA-3 transgenic and wild-type mice at different stages of disease progression. Panels B and C: Immunostaining for the CB_1_ receptor in the striatum of SCA-3 transgenic and wild-type mice at different stages of disease progression (bar = 50 μm and 10 μm, respectively). The arrows indicate the presence of CB_1_ receptor immunolabeling in corticostriatal terminals. In all cases, the values are expressed as the mean ± SEM of more than 6 mice *per* group, assessing the data using a Student’s t-test: *p<0.05, **p<0.01 *versus* wild-type animals.

We initially explored the changes in endocannabinoid signaling by studying CB_1_ receptor expression by qPCR, detecting a significant reduction in 32 and 56 week-old SCA-3 transgenic mice (Fig 3A). This reduction might be related to the loss of CB_1_ receptor-containing striatal projection neurons in SCA-3 transgenic mice, the cell bodies of which are located in the striatum (see above). Indeed, this response was similar to that described in Huntington’s disease, another polyQ disorder in which the striatum is preferentially affected [36]. Accordingly, we expected to find less CB_1_ receptor immunoreactivity in the cell bodies of striatal projection neurons in SCA-3 transgenic mice. However, we were unable to label these cell bodies in either SCA-3 transgenic or wild-type mice (Fig 3B), possibly because CB_1_ receptors localize predominantly to the terminals of neurons that arise in the globus pallidus and the substantia nigra, and not significantly to their cell bodies [37]. Conversely, we did detect CB_1_ receptors in terminals projecting to the striatum (see arrows in Fig 3B), almost certainly belonging to corticostriatal neurons [38]. Quantification of this immunolabeling indicated there were no changes in young, 16 week-old SCA-3 transgenic and wild-type mice, although there was notable reduction of immunoreactivity in SCA-3 transgenic mice at 32 and 56 weeks of age (Fig 3C). This reduction was not correlated with the reduction found in CB_1_ receptor mRNA in the striatum (Fig 3A), as the cell bodies for these neurons are located in cortical areas.

**Fig 4:**
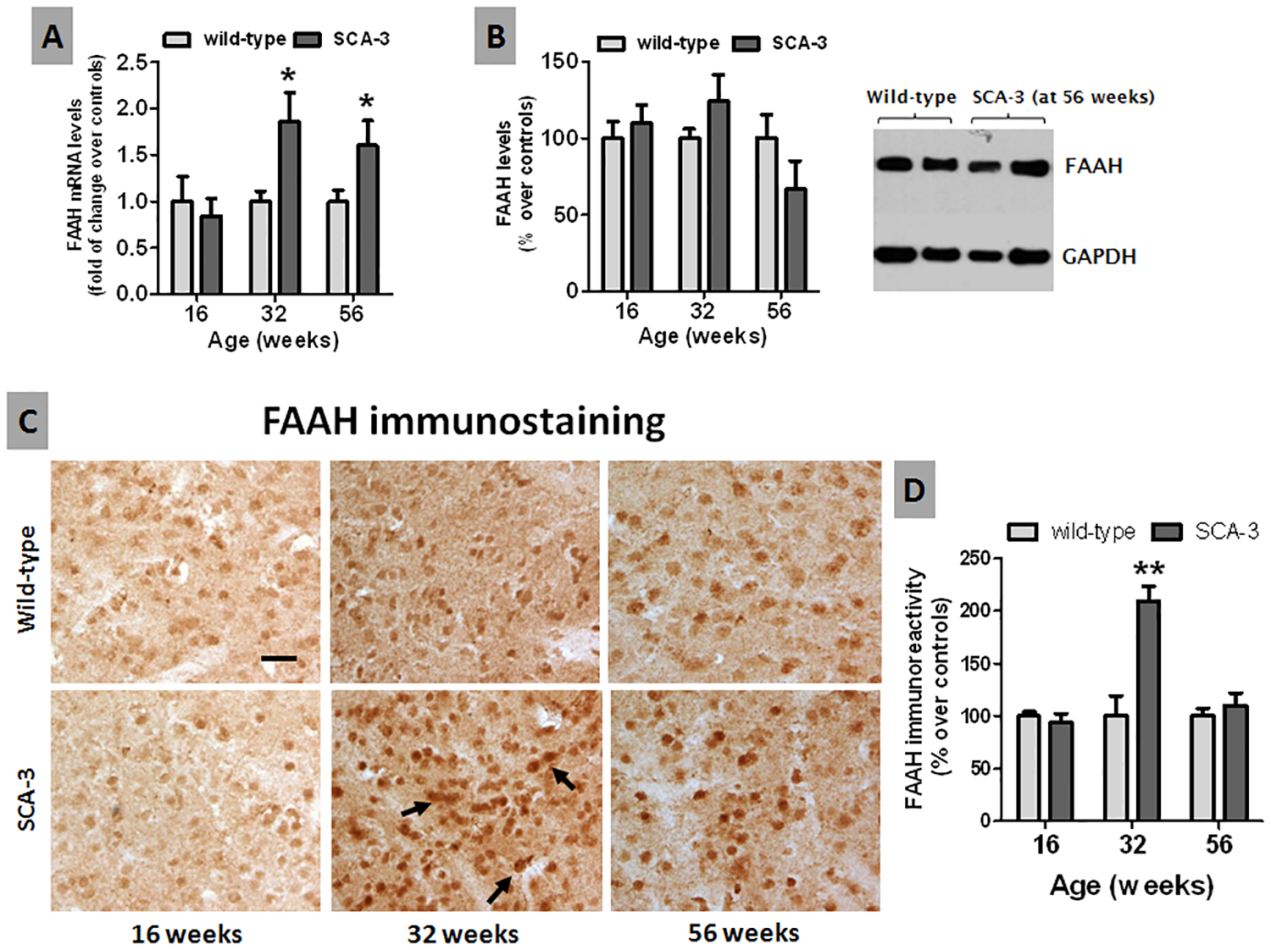
Histological and biochemical analysis of the FAAH enzyme in the striatum of SCA-3 transgenic and wild-type mice. Panels A and B: qPCR analysis and Western blot quantification (in a representative blot) of the FAAH enzyme in the striatum of SCA-3 transgenic and wild-type mice at different stages of disease progression. Panels C and D: Immunostaining for the FAAH enzyme in the striatum of SCA-3 transgenic and wild-type mice at different stages of disease progression (bar = 50 μm). Arrows indicate stronger immunolabeling in surviving striatal neurons of SCA-3 transgenic mice. In all cases, the values are expressed as the mean ± SEM of more than 6 animals *per* group, assessing the data using a Student’s t-test: *p<0.05, **p<0.01 *versus* wild-type animals.

We detected an increase in FAAH mRNA expression in the whole striatum at 32 and 56 weeks (Fig 4A), a change also seen in patients and animal models of Huntington’s disease [36]. However, no such increase was seen in western blots (Fig 4B). FAAH immunostaining demonstrated an intense signal in the cell bodies of striatal neurons (see arrows in Fig 4C), although there were no apparent differences in the cellular and subcellular distribution between SCA-3 transgenic and wild-type mice at 16, 32 and 56 weeks of age (Fig 4C). However, quantification of the immunostaining revealed elevated immunolabeling in SCA-3 transgenic mice at 32 weeks of age (Fig 4D), despite the deterioration of the striatal projection neurons described above (Fig 1A). Thus, as surviving neurons may express more FAAH (Fig 4A), this change may only be evident at local levels as it was not seen in western blots of the whole striatum (Fig 4B).

**Fig 5:**
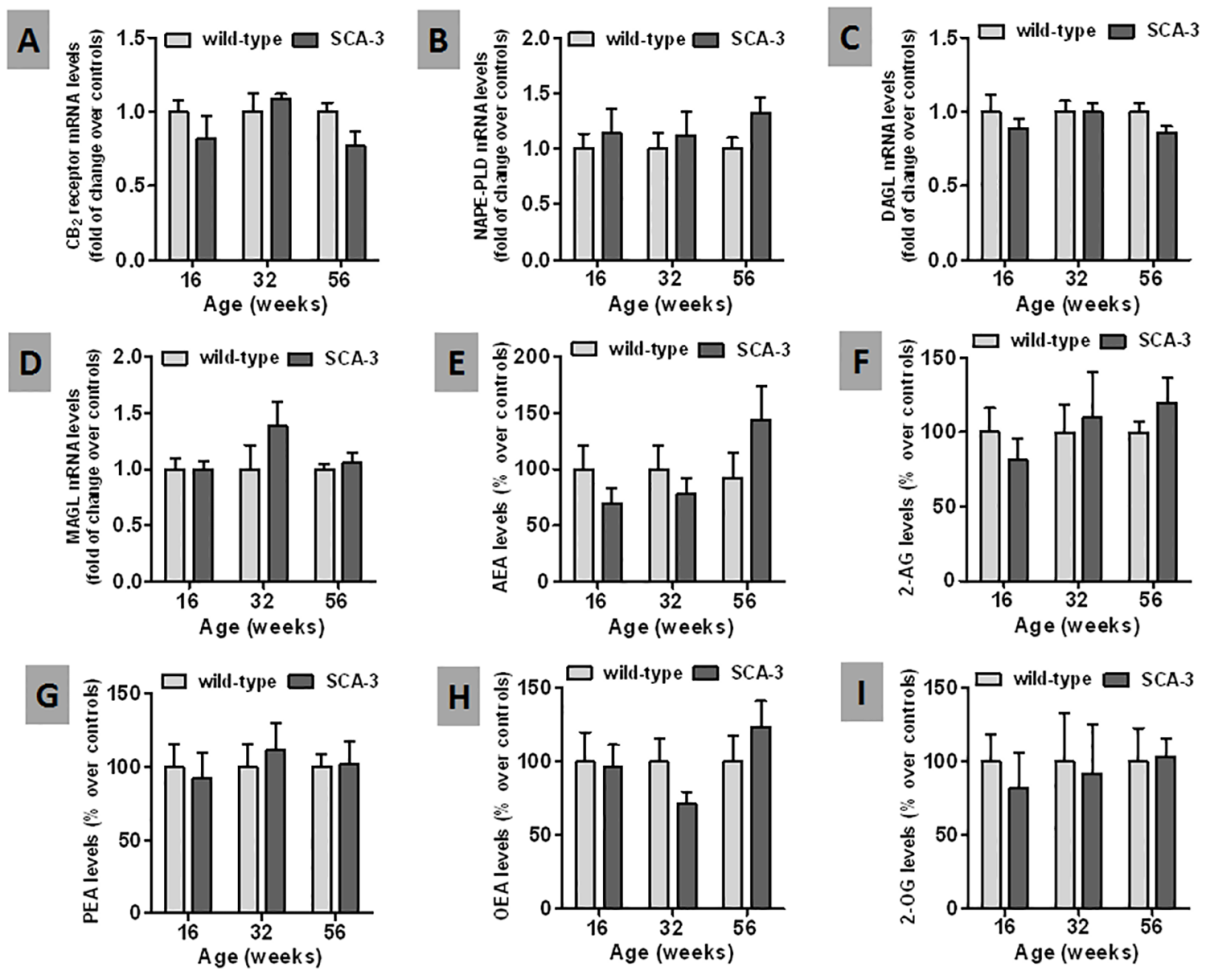
Biochemical analysis of endocannabinoid genes and proteins, and of related signaling lipids in the striatum of SCA-3 transgenic and wild-type mice. Panels A-D: qPCR analysis of the CB_2_ receptor and of the NAPE-PLD, DAGL and MAGL enzymes in the striatum of SCA-3 transgenic and wild-type mice at different stages of disease progression. Panels E-I: LC-MS analysis of the levels of anandamide (AEA) and its related congeners, palmitoylethanolamide (PEA) and oleylethanolamide (OEA), as well as 2-arachidonoylglycerol (2-AG) and its congener [2-oleylglycerol (2-OG), in the striatum of SCA-3 transgenic and wild-type mice at different stages of disease progression. In all cases, the values are expressed as the mean ± SEM of 5–6 subjects *per* group, assessing the data using a Student’s t-test.

Lastly, no differences were detected between SCA-3 transgenic and wild-type mice when other endocannabinoid genes were assessed at the three ages studied, including the CB_2_ receptor, NAPE-PLD, DAGL and MAGL (Fig 5A–5D). We also measured anandamide and 2-AG, as well as their related signaling lipids PEA, OEA and 2-OG, again failing to detect differences between SCA-3 transgenic mice and wild-type animals at the ages studied (Fig 5E–5I).

## Discussion

This study demonstrates that certain elements of the endocannabinoid signaling system (CB_1_ receptors, the FAAH enzyme) are altered in the striatum of SCA-3 transgenic mice, as previously found in other important brain structures affected in this type of ataxia (cerebellum and brainstem [17]).

The SCA-3 transgenic mice used in this study exhibited a broad-spectrum of motor anomalies that are comparable to the clinical symptoms found in patients. These included basal ganglia-related extrapyramidal signs like dystonia [17,28], which should *a priori* be the consequence of the selective deterioration of striatal projecting neurons that are also affected in the human pathology [24,25]. As in humans, neurodegeneration does not appear to be associated with marked glial activation or inflammatory events [21]. Rather, early alterations to glial glutamate transporters associate the degenerative process with elevated glutamate toxicity, consistent with our earlier observations in the brainstem nuclei [17]. Such toxicity was also found in other SCA-3 models [39] and it has been proposed to be the basis for the extension of neurodegeneration to extracerebellar structures in SCA-3 [20]. As such, the CMVMJD135 SCA-3 mouse model would appear to be useful for the study of extrapyramidal signs/basal ganglia deterioration in the progression of SCA-3, including the contribution of the endocannabinoid signaling system to this progression, the objective pursued herein.

Our goal was to determine whether endocannabinoid signaling is altered in the striatum of CMVMJD135 mice, possibly contributing to disease progression. This is indeed the case, as our findings are consistent with a possible reduction in CB_1_ receptor signaling with elevated FAAH levels in this structure, both events that are compatible with enhanced excitotoxic damage. The nature of such altered endocannabinoid signals is clearly dependent on the cellular context, as the changes were not exactly the same in the striatum as in the cerebellum and brainstem [17]. In addition to contributing to disease progression, the changes in endocannabinoid signaling in SCA-3 transgenic mice may be associated with the appearance of specific neurological symptoms like the loss of motor coordination observed in these mice. Such symptoms were previously related to the elevated CB_1_ receptor-mediated signaling in the Purkinje cell layer, predominantly in terminals of the basket cells [17]. Along similar lines, the reduced CB_1_ receptor signaling found in the striatum may be related to the dystonia (clasping behavior) described in these mice [17], particularly given the relevance of the basal ganglia circuits in controlling dystonic responses [34,35]. However, recent data related the onset of dystonia to the malfunction of other CNS structures, including the cerebellum [40], making it possible if this is also related to the altered endocannabinoid signals in the cerebellum.

Such alterations to CB_1_ receptor signaling could be pharmacologically corrected using direct agonists of this receptor or even by elevating the endocannabinoid tone by inhibiting endocannabinoid inactivation (e.g. FAAH/MAGL inhibition). Such an approach should attenuate dystonia, as documented in a few pharmacological studies on dystonic patients [41] or experimental models [42]. While this may relieve these symptoms, our aim was to obtain a more effective and sustained alleviation of these symptoms by controlling disease progression through cannabinoid-based therapies [3]. In this sense, our data indicate that the activation of CB_1_ receptors may also be beneficial to avoid the loss of striatal projection neurons, as described previously in murine models of Huntington’s disease [36]. In both cases, alleviating dystonia or preserving striatal neurons, the therapy aims to correct the changes in endocannabinoid signaling by enhancing CB_1_ receptor function, assuming that these changes represent a maladaptive response that contributes to disease progression. This appears to be the case for the elevated FAAH levels found in the striatum, which would also be a maladaptive response possibly contributing to disease progression through excessive endocannabinoid degradation. In this sense, it may be better to selectively inhibit the FAAH enzyme in order to enhance the levels of endocannabinoids and their protective function. In both cases, enhancing CB_1_ receptor signaling or inhibiting FAAH enzyme would address the changes in both targets that may contribute to this pathology.

The neuroprotection elicited by cannabinoids may also involve an enhancement of specific responses that represent adaptive mechanisms aimed at restoring neuronal integrity and homeostasis in the face of neurotoxic insults [43]. We expect this may be the case of CB_2_ receptor-mediated signaling, given that it is up-regulated in an attempt to preserve neurons against inflammatory events in most neurodegenerative disorders [2,4], including SCAs when *post-mortem* tissue from patients is studied [15]. However, we were unable to find any evidence of such events in the striatum of these SCA-3 transgenic mice, as occurs in the cerebellum and brainstem [17] where CB_2_ receptor levels also remain unaffected. We assume that the lack of a CB_2_ receptor response may reflect the relatively limited glial reactivity found in the affected CNS areas in this model [28].

Another adaptive response frequently elicited by endocannabinoid signaling in response to damage is an elevation in the generation of endocannabinoids. This has been described for different chronic neurodegenerative disorders [1], yet as in the other CNS structures investigated previously [17], we did not find elevated endocannabinoid levels in the striatum of SCA-3 transgenic mice. In fact, given the elevated FAAH immunostaining in specific striatal neurons the expected local reduction of these signaling lipids was not evident in Western blots, most likely due to the use of the whole striatum. Nevertheless, such a response, elevated FAAH activity/local reduction of endocannabinoids, is likely to contribute to disease progression rather than being an adaptive protective response.

## Conclusions

Our results from SCA-3 transgenic mice confirm that certain elements of the endocannabinoid signaling system are altered in the striatum, as previously found in other important brain structures affected in this type of ataxia (cerebellum, brainstem [17]). Such changes may contribute to the pathogenesis of this condition by aggravating the influence of different neurotoxic stimuli in these mice (e.g. excitotoxicity). Hence, a pharmacological manipulation that corrects the changes in endocannabinoid signaling could be an interesting objective for future research.
